# Connecting single-cell properties to collective behavior in multiple wild isolates of the *Enterobacter cloacae* complex

**DOI:** 10.1371/journal.pone.0214719

**Published:** 2019-04-04

**Authors:** Sean Lim, Xiaokan Guo, James Q. Boedicker

**Affiliations:** 1 Department of Physics and Astronomy, University of Southern California, Los Angeles, California, United States of America; 2 Department of Biological Sciences, University of Southern California, Los Angeles, California, United States of America; University of Illinois at Urbana-Champaign, UNITED STATES

## Abstract

Some strains of motile bacteria self-organize to form spatial patterns of high and low cell density over length scales that can be observed by eye. One such collective behavior is the formation in semisolid agar media of a high cell density swarm band. We isolated 7 wild strains of the *Enterobacter cloacae* complex capable of forming this band and found its propagation speed can vary 2.5 fold across strains. To connect such variability in collective motility to strain properties, each strain’s single-cell motility and exponential growth rates were measured. The band speed did not significantly correlate with any individual strain property; however, a multilinear analysis revealed that the band speed was set by a combination of the run speed and tumbling frequency. Comparison of variability in closely-related wild isolates has the potential to reveal how changes in single-cell properties influence the collective behavior of populations.

## Introduction

Linking collective behaviors of bacterial populations to the rules followed by constituent cells is an important challenge in biology [1–3]. An intriguing manifestation of collective behavior exhibited by some motile species is the formation of a “swarm band,” a conspicuous grouping of cells into a migrating region of high cell density [4, 5]. In either semisolid or liquid media, the band forms and propagates as a result of the self-organization of cells that dynamically respond to gradients in chemoattractants or changes in cellular energy levels [6–11]. Many studies on the mechanisms of band formation also attribute the band’s behavior, a macroscopic property, to the motility dynamics of single cells [4, 8, 12]. Several studies have examined how individual motion of cells relates to properties of the collective [13, 14]. However, there have been limited experimental studies investigating differences in collective behavior between closely related strains of bacteria [15].

Genetic variations between strains have been previously shown to impact macroscopic behavior by acting on several motility subsystems [16, 17]. To move, bacteria rely on a series of “runs” (unidirectional swimming) and “tumbles” (sharp reorientations) to translocate themselves in space using helical, whip-like structures called flagella [12, 18, 19]. Food consumption and signal production generate molecular gradients in environments that were initially uniform. Cells detect these gradients and perform a biased random walk in the direction of a gradient [20]. In this way, cell movement and activity potentially drive populations to chemotactically self-organize in space [9, 21]. Thus, natural genetic variation among wild-type strains in their growing and swimming faculties may result in varying degrees of macroscopic migratory behavior. A recent study by Fu, X., Kato, S. et al. has shown variability in tumbling bias within genetically identical population can result in distinct migration speeds [22]. However, studies are often limited to single species in *Enterobactericae*, such as *Escherichia coli* or *Salmonella typhimurium*, for which most experiments and theoretical models on collective motility behavior have been performed for [5, 8, 23–27]. Examination of natural, subtle variation in both collective and single-cell properties should yield new insights as to how these two scales of behavior are interrelated.

Here, we examined self-organization manifesting itself in the band formation of wild isolates of another member of the *Enterobactericae* family. By screening for wild coliform bacteria in freshwater samples, we identified 7 strains of bacteria belonging to the *Enterobacter cloacae* complex (Ecc) that exhibited self-organized, collective migration. Each strain formed a band several hundred microns in width composed of a dense concentration of swimming cells on nutrient-supplemented semisolid agar [5]. *Enterobacter cloacae* are rod-shaped, facultative anaerobic bacteria belonging to the coliform group [28]. Strains of the Ecc are often implicated in infectious diseases in agriculture and healthcare settings [29–32]. Across wild strains, we observe a diversity of band propagation speeds, ranging from 2 mm/hr to 5 mm/hr. Due to the natural variability of band propagation speeds, these wild strains were an ideal system to examine how variability in collective behavior stems from differences in single-cell behavior. Ecc strains, being a non-laboratory model strain, also offer the opportunity to test whether our understanding and models of bacterial collective migration can be extended to bacteria beyond *Escherichia coli*. By characterizing each strain’s run-and-tumble behavior and growth rates, we identify a range of individual properties underlying the collective motion. These strains were perturbed by the exclusion of a single amino acid from the migration media to observe the change in both collective and single-cell behaviors. Surprisingly, we found a heterogeneous response, with some strains reducing their band speeds more than others. Together, the analysis of multiple strains under these two conditions revealed run speeds and tumbling frequency together set the speed of the swarm band.

## Results

### *Enterobacter* wild isolates form swarm bands of varying propagation speeds

Traveling waves of high cell density, swarm bands, have been observed in other members of the *Enterobactericiae* family such as *Salmonella typhimurium* and *E*. *coli* [5, 23, 26] (see Fig A in S1 File for growth curves). To compare formation of swarm bands in closely related strains, we isolated a variety of bacteria strains from freshwater sources. To select strains of the *Enterobactericiae* family, we utilized HardyChrom^™^ ECC selection plates to collect multiple strains of the *Enterobacter cloacae* complex from the wild. Each isolate was screened for band formation and the 16S rRNA region was sequenced.

**Fig 1:**
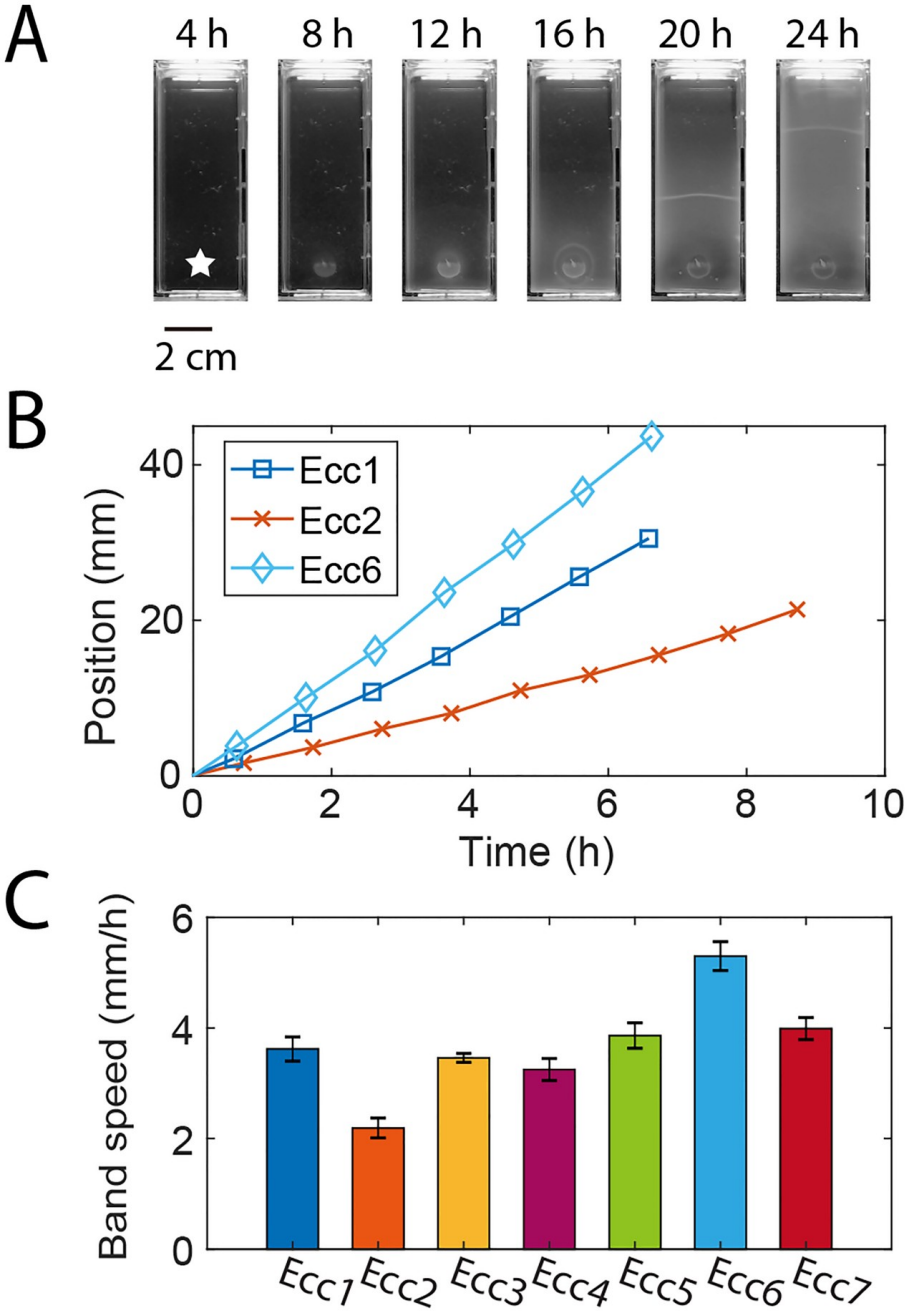
Propagation of the bacterial swarm band formed by strains of the *Enterobacter cloacae* complex in semisolid (0.26% agar) migration media. (A) The bacterial swarm band begins at the inoculation point (as denoted by the star) and travels along the channel. See also S1 Movie. (B) A representative position vs. time plot of the band from 3 different Enterobacter strains. The band speeds are nearly constant and differ from strain to strain. (C) Band speeds of each strain represented as mean ± SEM (n≥5 independent experiments). See also Fig B panel C in S1 File.

Following previous studies, we inoculate 10μL of bacteria culture grown overnight onto migration media, an M9 Minimal Salts-based semisolid agar (0.26%) [5, 23, 26]. The agar concentration of our media is slightly lower than the minimum concentration (0.3%) that required for surface swarming motility of bacteria [33]. Agar media at 0.26% is a porous medium whose matrix presents cells with obstacles for movement [8]. We observe bacterial migration over 2 days at 25°C using a custom setup (Fig B panel A in S1 File). Before migration commences, the cells grow into a dense colony at the point of inoculation (Fig 1A, S1 Movie). About 12–18 hours after inoculation, the swarm band coalesces at the center of the colony and begins to radiate outward as a ring [5]. The ring is visible by eye and can be tracked easily without magnification through time-lapse imaging (Fig B panel B in S1 File). Starting out slowly, the ring then attains a near-constant propagation speed held over the next 1–2 days (Fig 1B). The band maintains its shape until it has completely propagated to the other side of the well. Our custom setup allowed for rapid simultaneous quantification of the migration patterns of several bacterial strains (Fig B panel A in S1 File).

We turned our attention to assess the variation of band speeds in wild *Enterobacter*. To compute average velocity for a specific strain, band positions were collected every 30 minutes using manual tracking starting 1.5 cm away from the inoculation point. A linear fit gave the band velocities, and in replicate experiments, the band speed was reproducible within about 1 mm/hr (Fig B panel C). For the several isolates compared, the average of the band speeds ranged on average from 2 mm/hr to 5 mm/hr, which is on the order of 1 cell length per second (Fig 1C). Such variation between strains was the motivation for us to subsequently investigate the motility and growth of each strain and their relationship to band speed.

### Band Speed is not correlated with any individual strain property

To explore the correlations between the observed variation in macroscopic band speed and several strain properties, we analyzed both single-cell motility and the growth rate of each strain. For motility, we used time-lapse microscopy on cells in a microfluidic chamber (C-Chip DHC-S02-2, INCYTO) where we recorded the run-and-tumble behavior of each strain. Microfluidic devices with thin chambers have previously been used to capture the motility of cells by constraining cell motion to a plane and to prevent net movement of fluid, allowing for longer observation times [17, 34]. The cells, after a 100-fold dilution from overnight culture, were grown at 25°C to early exponential phase in M9 medium. Cells were diluted to a density which gives approximately 300 cells/mm^2^ in the chip and then injected into the chip’s microchamber with a depth of 20 μm. 5-minute movies of the cells were recorded in phase contrast illumination with a 40X microscope objective.

**Fig 2:**
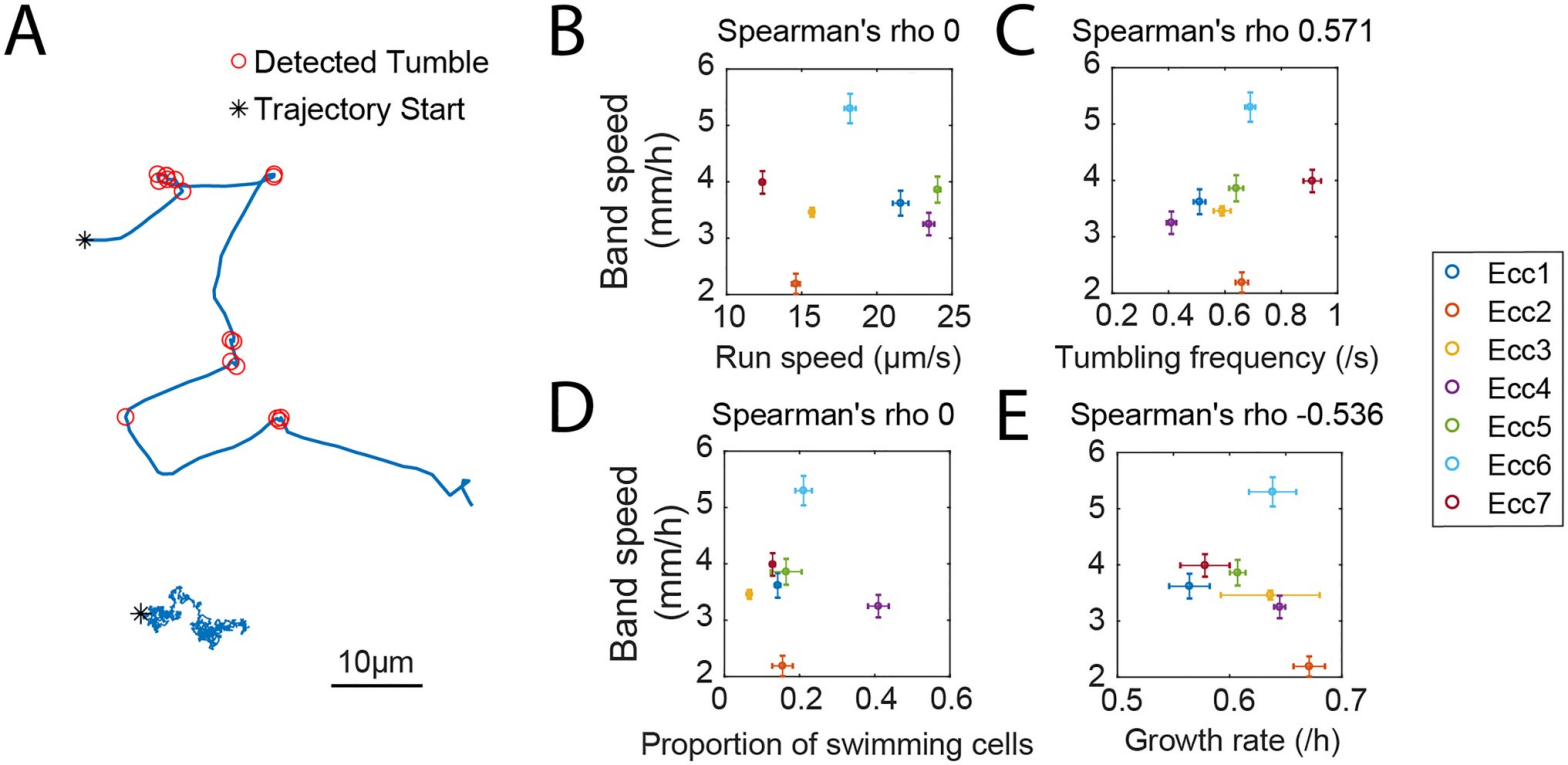
Strain properties and their correlations with band speed. (A) Example trajectory showing tumbling events. The bottom trajectory is from a non-swimming cell. See also Fig C panel A in S1 File. Correlations are shown between the band speed and (B) the run speed, (C), the tumbling frequency, (D) the proportion of swimming cells, and (E) the growth rate. Plotted is the mean band speed ± SEM, with n≥5 independent experiments. Each strain property is the mean ± SEM, with n = 3 independent measurements. See also Fig D panel A in S1 File.

We characterized the run-and-tumble behaviors to each strain (Fig 2A) [4, 8, 12, 17]. Single-cell trajectories were compiled using the TrackMate plugin of the image processing program ImageJ [35]. We collected trajectories from 3 separate areas of the microchamber for each strain. Trajectories less than 5 seconds long were discarded, giving more than 140 trajectories per location. Following analysis of trajectories presented in [34], we measured the angular velocity of each cell over time. We detected tumbling events of cells using an algorithm comparing local maxima to neighboring minima of angular velocity (see Trajectory Feature Detection in Materials and Methods). A run event was defined as any trajectory segment between two tumbling events that was at least 0.5 seconds long.

To quantify strain properties, we calculated the run speed as the average speed of all run events captured in a video. The tumbling frequency is the inverse of the average run time between two tumbling events [34] (Fig C panel A and Fig D panel B in S1 File). We utilized Spearman’s rho rank correlation coefficient to assess how monotonic the quantitative connections are between a strain property and the band speed [36]. With the critical value of Spearman’s rho 0.786 for 7 samples at 5% significance level, Fig 2B and 2C show no strong correlation between the run speed and the band speed, or between the tumbling frequency and band speed. Therefore, an examination of one motility property by itself was not adequate to explain increasing band speeds.

We found similar weak trends between band speeds and other properties of the strains that may also contribute to the band speed. Previous studies have shown differences in the fraction of motile cells between strains of the same species [37, 38]. Theories on collective behavior also propose the key role of the mixture of non-swimming and swimming cells in governing collective motility [39–41]. We found a subpopulation of non-swimming cells for each strain. The proportion of swimming cells was calculated as the average amount of cells observed with an average speed larger than 0.32 μm/s, which is a tenth of their body length per second. The non-swimming cells (with average speed less than 0.32 μm/s) are cells that are not be able to swim efficiently in liquid media (Fig C panel B in S1 File). Although the difference between swimming and non-swimming cells is not clear, previous studies have reported a few mechanisms of motility regulation in bacteria. Motility in some species is regulated by environmental conditions [42–44], and the production of flagella can be regulated by a genetic switch [45]. Non-swimming cells were not stuck to chamber surfaces, as all cells migrated multiple cell lengths during the data acquisition period. Strains assessed here had low proportions of swimmers, ranging from less than 10% to a little over 40% (Fig 2D). However, the band speed was not correlated with fraction of swimmers. Although non-swimming cells would contribute to the shape and dynamics of chemotactic gradients, it seems reasonable that non-swimming cells would not strongly influence the band speed. Many studies have shown that fraction of motile bacterial cells is usually low (about 10%) in natural environment, and may vary a lot (5% to 70%) depending on environmental factors [12, 42, 44, 46, 47].

Cells growth rate has been shown to contribute to the band speed [41], and a standard model of band formation, Keller-Segel model, predicts a dependence of band speed on growth rate (Fig F and Table A in S1 File). We measured the exponential growth rates of all *Enterobacter* strains in the same migration media and temperature as the band speed experiments. Although varying between strains, growth rate also lacks a monotonic relationship with increasing band speeds (Fig 2E). We also correlated every strain property we measured to each other and also found no striking monotonic relationships (Fig D panel A in S1 File, blue scatter). In summary, these results suggest that no single single-cell property is dominant in setting the band speed.

### Band speed and strain properties are perturbed by exclusion of methionine from media

As a variety of strain properties underlie band propagation speeds, we then aimed to perturb these properties in each strain. Methionine or its metabolites are believed to be involved in tumbling process of bacterial cells. Previous studies have characterized cells grown without methionine and found cells have a reduced frequency of tumbling in chemotactic response [48–50]. Apart from cell motility, methionine, as a required amino acid for growth, can also influence the growth rate. Synthesizing methionine has been shown to be an energy-consuming process that delays growth [51]. Given methionine is a modulator of both motility behavior and growth, we removed methionine from the media to determine how this perturbation would impact both single-cell properties and the band speed of each strain.

**Fig 3:**
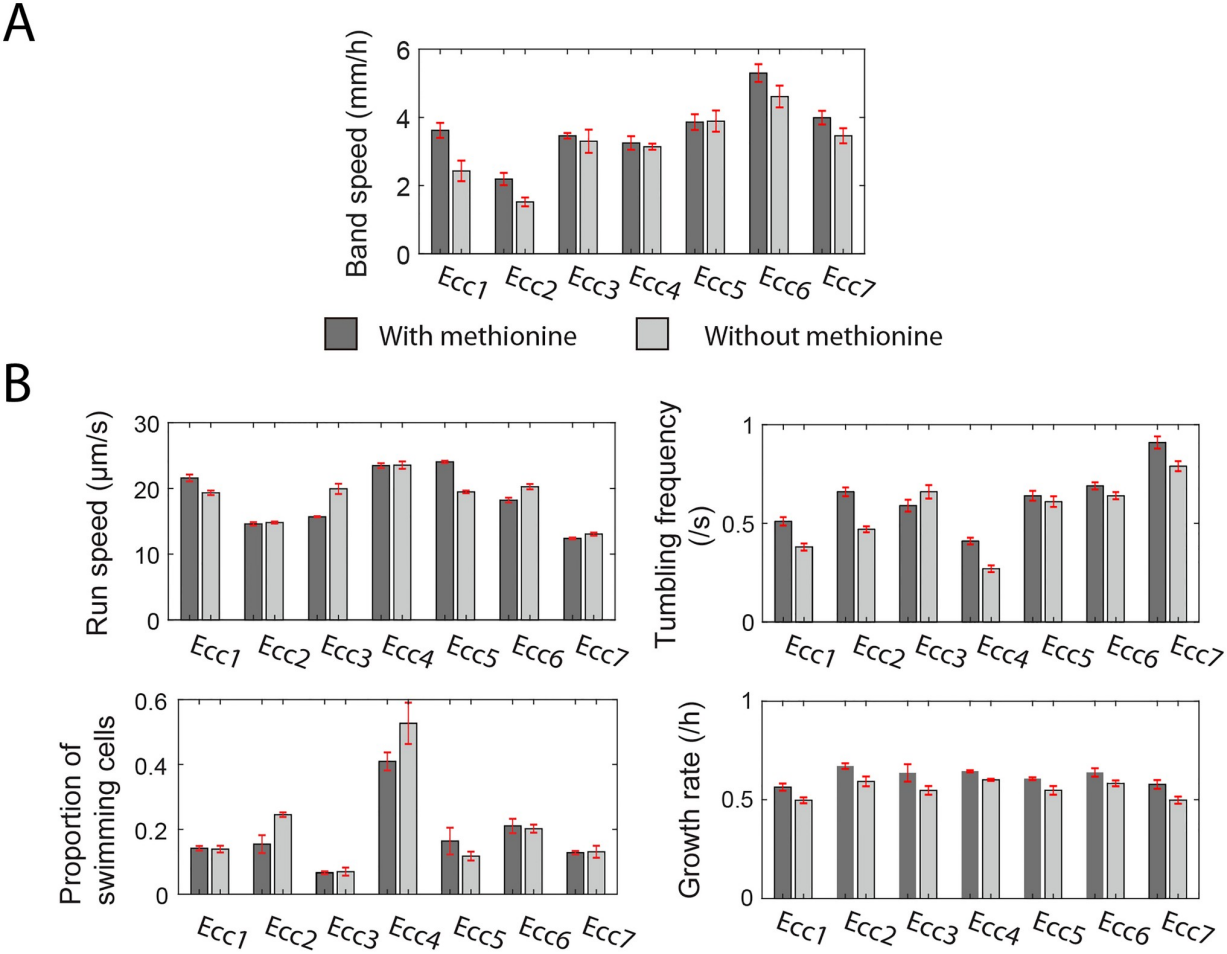
Removal of methionine from migration media altered both strain properties and band speed. (A) The band speeds of all *Enterobacter* strains shown in Fig 1 are compared to the band speeds when cells are grown on migration media without methionine. Band speeds of each strain represented as mean ± SEM (n≥3 independent experiments). (B) Single-cell motility properties and exponential growth rates also changed upon removal of methionine from the media. Strain properties represented as mean ± SEM (n = 3 independent measurements).

We conducted migration experiments as shown in Fig 1A but without methionine added to the migration media. The band speeds of *Enterobacter* strains in an initially methionine-free environment are compared to the band speeds with methionine as shown in Fig 3A. The response to the removal of methionine was heterogeneous among the seven wild isolates; some band speeds decreased to a variable extent while others remained unchanged (Fig 3A). Simulations based on Keller-Segel model reveal similar changes of band speed as observed in experiments (Fig F in S1 File). We then assessed single-cell swimming behavior and growth rates in the absence of methionine to better understand which of these properties may be associated with the alteration of band speed (Fig 3B). There is no universal pattern of the effect of methionine on the run speed and proportion of swimming cells of each strain. However, 6 out of 7 *Enterobacter* strains tumble less frequently in environment without methionine, and growth is slower for all isolates in the absence of methionine (Fig 3B). Therefore, comparing Fig 3A and 3B, the growth rate and tumbling frequency might seem to be a positive contributor to the band speed as their shifts are most similar.

Although band speed shows no significant correlation with growth rate as shown in Fig 2E, in the absence of methionine, all growth rates decrease whereas band speeds also decreased by variable amounts. Nevertheless, the shifts of other strain properties are not monotonically associated with the shift of band speed.

### Run speed and tumbling frequency in combination determine band speed

We then considered the band speeds of strains in environments with and without methionine as response variables and the four strain properties as predictor variables. To examine whether combinations of predictor variables were correlated with the response variable, we applied multiple linear regression (MLR) to infer the regression coefficient of each property. Since different properties have different units and magnitudes, we defined the rescaled sensitivity as the regression coefficient of a property multiplied by the average value of that property of all strains analyzed in conditions, both including and excluding methionine.

**Fig 4:**
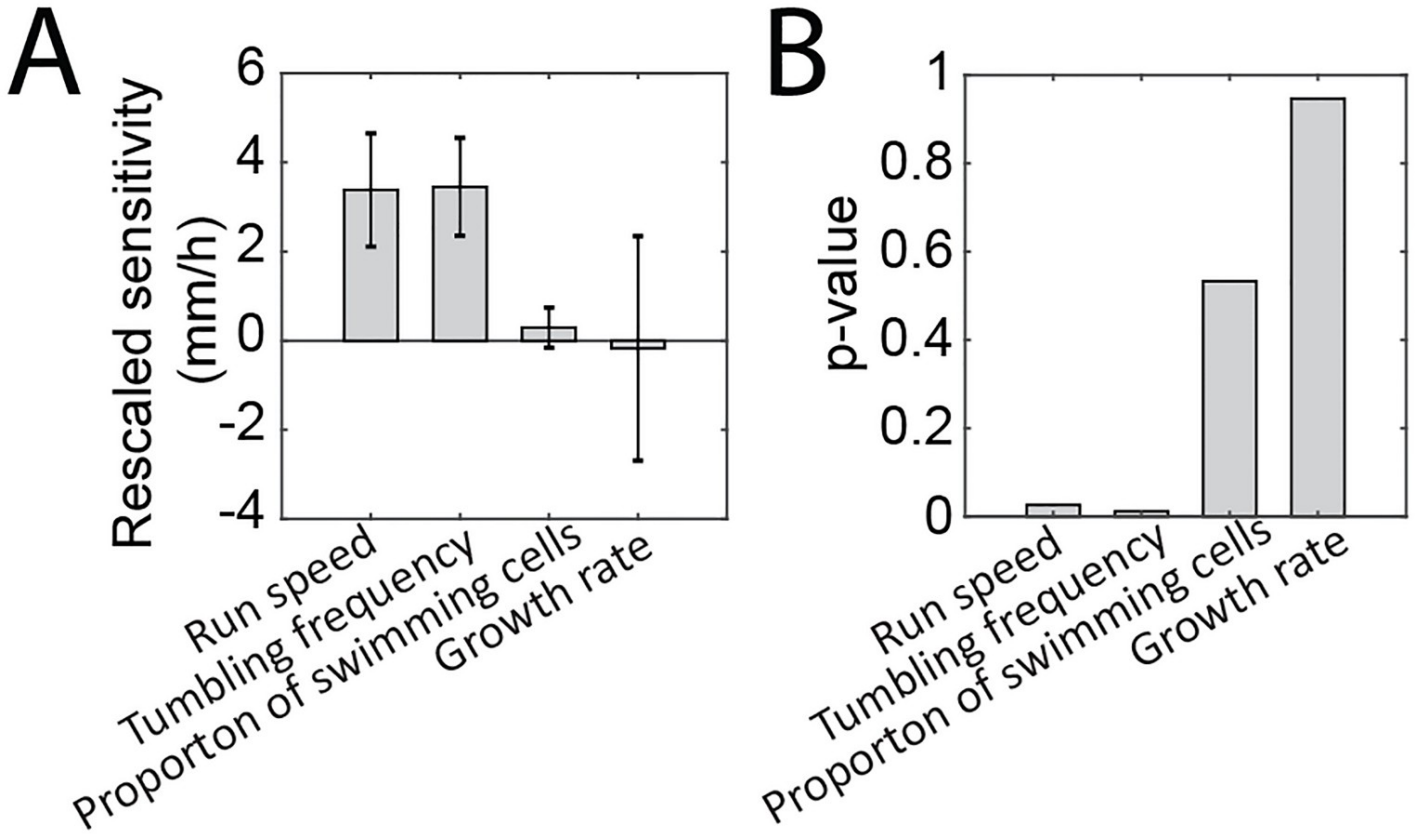
Multiple linear regression reveals band speed is connected with run speed and tumbling frequency. (A) The rescaled sensitivity found from multiple linear regression is the regression coefficient of the single-cell characteristic multiplied by the average value of that characteristic of all strains. The results indicate run speed and tumbling frequency are the dominant factors that influence the band speed. Error bars indicate rescaled standard error. (B) The p-values for F statistic of all characteristics. The values are 0.019, 0.016, 0.762, and 0.929 for run speed, tumbling frequency, proportion of swimming cells, and growth rate respectively.

Fig 4A shows the rescaled sensitivities of run speed, tumbling frequency, proportion of swimming cells, and growth rate. Only two parameters were significantly non-zero, run speed and tumbling frequency (Fig 4B). This has also been verified by simulation based on Keller-Segel model (Fig F in S1 File). The p-value for each property was calculated using a null hypothesis that the corresponding coefficient is equal to zero. Both run speed and tumbling frequency were positively correlated with band speed and were nearly equally predictive of the band speed.

**Fig 5:**
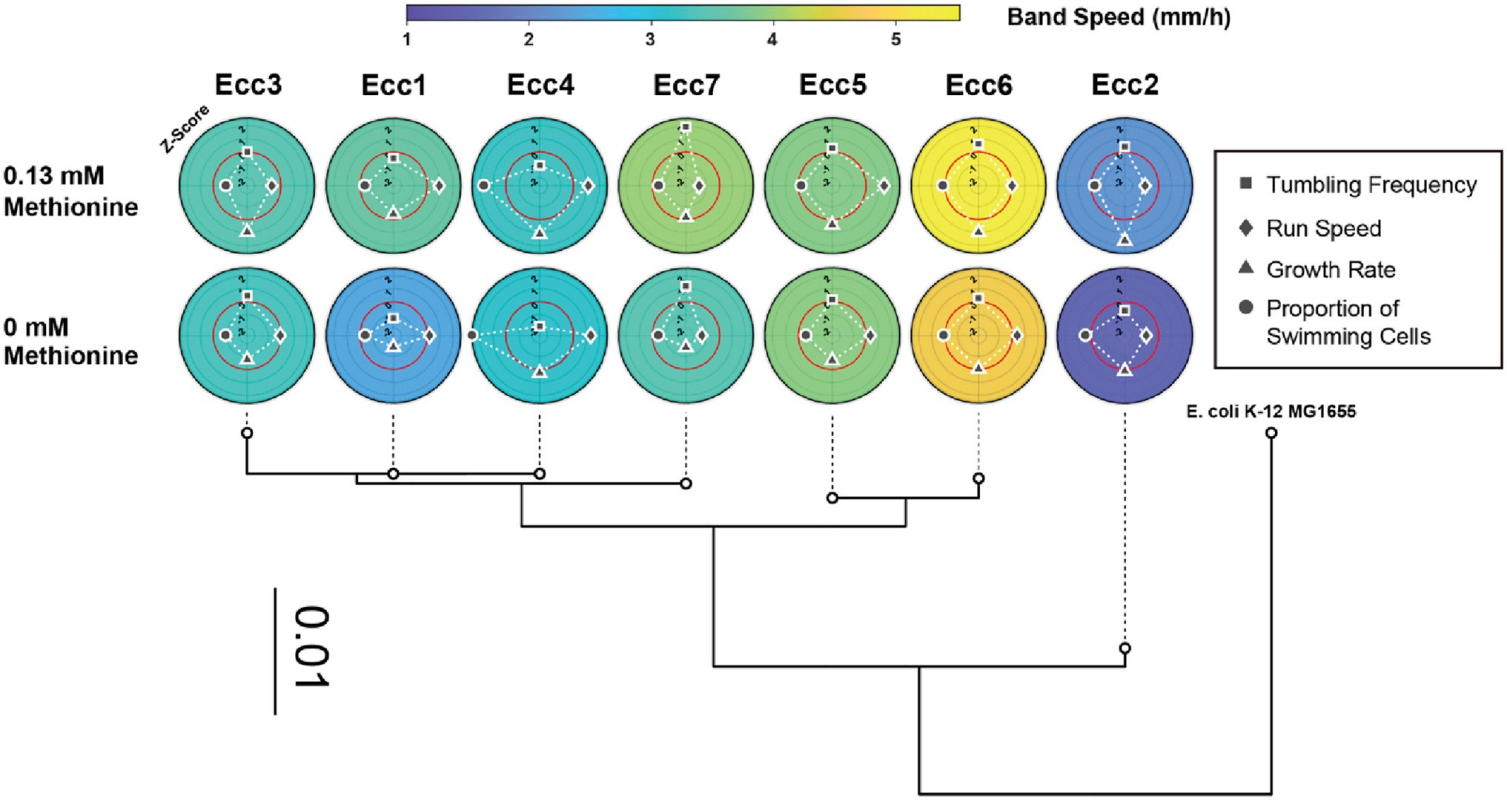
Related *Enterobacter* strains have distinct combinations in strain properties and band speeds. A spectrum of strain properties underlies the band speed in nutrient-supplemented semisolid media (Row 1: with methionine, Row 2: without methionine). Each strain’s property has an associated Z-score from the mean value of that property across the 7 strains analyzed in this study. The red circle represents a Z-score of 0 (no deviation from the mean), and each gray concentric circle is an increment of a Z-score of 1. The band speed is the color of the plot. Below, the phylogenetic tree from 16S sequences are plotted. *E*. *coli* K-12 MG1655 is provided as a reference outgroup. The scale bar represents 0.01 changes per base pair.

Given the rescaled sensitivities and p-values of all properties, the run speed and tumbling frequency both significantly contribute to the band speed to a similar extent. This conjecture is supported by a linear combination of both properties with the coefficients found in MLR to the band speed (Fig E panel B in S1 File). The proportion of swimming cells and the growth rate, even in combination with other strain properties, were not as strong as the combination of run speed and tumbling frequency (Fig E panel B in S1 File). This analysis shows that the band speed is equally influenced by two single-cell properties, run speed and tumbling frequency, and that the strain-to-strain variability in both parameters modulates the collective motility of populations. The nearly equal contributions of run speed and tumbling frequency also explains why the band speed is not strongly correlated with either property, as high run speed might be randomly paired with low tumbling frequency, and vice versa. Amongst the 7 strains studied, similar band speeds were detected in strains with different combinations of run speed and tumbling frequency, as shown in Fig 5. Fig 5 also shows that band speed was similar for closely related strains, as measured by 16S rRNA comparison. Closely related strains Ecc5 and Ecc6 had fast band speeds, while the more distantly related strain Ecc2 had the slowest band speed.

## Discussion

Here we examine the band speed within a set of closely related natural isolates of the *Enterobacter cloacae* complex (Ecc). The band that propagates in semisolid media has been previously examined experimentally and modeled in several species, notably *E*. *coli* and *Salmonella typhimurium* [5, 24, 52, 53]. Here for the first time we have described the band speed in the Ecc. Unlike the collective behavior of some *E*. *coli* or *Salmonella typhimurium*, this band does not break up into smaller “droplets” or “spots,” which are regions of bacteria aggregation [23] (Fig 1A). The band resembles the radiating structure observed by Adler, for which he associated with propagating glucose or oxygen gradients [5]. These bands move at speeds of 2 mm/hr to 5 mm/hr, which is comparable to strains in other migratory studies in semisolid agar [53] (Fig 1B). This means the population of cells perform a collective unidirectional motion at about 1 cell length per second. We demonstrate that a strain with both high run speed and tumbling rate may increase how fast strains move as a group, which may help to understand migration in environments such as host tissue or soil [54–56].

Another novel report is our comparison of varying band speeds formed by closely-related wild strains (Fig 1C). As the traveling band is composed of a large number swimming bacterial cells, the study of single-cell motility from a microscopic perspective is essential for a better understanding of the band speed. Despite having high genetic similarity, the differences in band speed across strains varied by more than a factor of 2 (Fig 5). These phenotype differences have previously been elucidated by genetic knockout studies in a single strain [16, 17, 24], revealing that collective motility is a complex phenotype influenced by many genes and microscopic properties of the strain. By comparing variability of both band formation and single-cell level properties across several closely-related wild strains, we sought to better understand how the band speed is set by the behavior of individual cells.

Intrigued by the natural variability of band speed, we took an in-depth look at the properties of the different Ecc strains. The strain properties we measured were the average run speed, tumbling frequency, proportion of swimming cells, and exponential growth rate. Runs, tumbles, and number of swimming cells are quantities that pertain to single-cell motility, and we obtain these quantities from measuring individual cells in a population. Upon comparison, no obvious correlation was found between the band speed and any one strain property, despite recent study showing that the band speed is negatively correlated with tumbling frequency [22], suggesting that the band speed is a complex property and dependent on multiple factors (Fig 2B–2E and Fig E panel A in S1 File). Multiple linear regression revealed that a combination of both run speed and tumbling frequency is the most significant predictor of the band speed (Fig 4B). In our strains, no individual characteristic predicted the rank ordering of the band speeds, possibly because in the relatively small number of strains uncorrelated variance of tumbling frequency and run speed obscured trends that may be visible in larger datasets or experiment with synthetic systems in which only a single factor was varied at a time. In the wild strains, variability of multiple factors between strains and the fact that two characteristics strongly contributed to setting the band speed prevented single factor correlations from identifying key parameters.

Here we focused on properties previously shown to resolve single-cell motility differences between closely-related strains (19, 36, 49). Other strain properties not measured here may strongly contribute to collective behavior, such as the average tumbling angle, chemotactic adaptation rate, as well as intracellular parameters such as metabolic rate, pH, and membrane potential (62–67). Further studies are needed to connect the band speed to additional single-cell properties to determine if additional strain properties are needed to predict the band speed in other systems. In addition, as we only examined 7 *Enterobacter* wild strains, whether our conclusions and approaches can be applied to other species remains to be tested.

Previously, it was found that collective migration was proportional to tumbling rate, which is consistent with our findings [8, 24, 57]. Our results emphasize that, for complex behaviors such as band formation, analysis within a variable set of related cell types may be necessary to resolve relationships between single-cell and collective behaviors. High run speed and high tumbling rate may facilitate cells to successfully “reverse-out” of and escape such dead-ends in gel matrices, allowing swimming cells to collectively move at a higher rate [8, 53, 58]. Tumbling frequency is also an essential component of chemotaxis as shown by studies with *E*. *coli* [59, 60]. In certain ranges, higher basal tumbling frequency or run speed may allow cells to more efficiently climb chemical gradients [14, 61, 62].

It should be noted that, in previous proposed physical models, growth rate is often positively associated with faster colony expansion [63–65]. Indeed, the exclusion of methionine attenuated growth rate for all strains while also reducing the band speed of some strains (Fig 3). It is not clear why in our strains growth rate was not significantly correlated with the band speed. Naively, one might expect that an increased growth rate would increase the rate at which chemoattractant gradients change over time, thereby increasing the band speed. Perhaps the larger variation in the tumbling frequencies and run speeds (both varying over a factor of 2) as compared to growth rate differences (varying by 20%) led to growth rate not being a dominant factor in setting in the band speed of these strains. Cells found in front of the high density band, as seen in S1 Movie, would help shape the chemotactic gradients responsible for the dynamics of the high density swarm band. This leading edge of cells is predicted by the Keller-Segel model, although the connection between the leading edge of cells and the band speeds was not examined here. The Keller-Segel model does predict that growth rate should help set the band speed, with measurable band speed differences even at 20% variation (Fig F in S1 File). The attempt to relate single-cell characteristics to the band speed using the Keller-Segel model revealed that the model could not accurately predict the wide variation of band speeds and did not capture the true sensitivity of the band speed to single-cell properties. Although the Keller-Segel model was able to reproduce the formation of the swarm band, even predicting the speed of the band within a factor of two of experimental measurements, the model could not predict the finer details of the seven bacterial strains measured in experiment. Part of these discrepancies could lie in the fact that model parameters were largely based on previous work with *E*. *coli*, whereas the experimental measurements here use seven wild variants of a different bacterial strain, *Enterobacter cloacae*, which has not been characterized as extensively as *E*. *coli*. For example, the chemotactic response of these strains to individual media components was not quantified. Here, the model makes the assumption that the added amino acids are the chemoattractant rather than the glucose that was added as part of migration media. The model was extended to include the influence of gel pore size on the restricted movement of bacteria, see Fig F panel B in S1 File, however even in the small pore size limit the range of observed band speeds and their rank ordering could not be reproduced by model simulations. Although the version of the model implemented here could not account for the experimentally measured variation in the band speeds in the wild strains, it is not clear that agreement with a model of this form would be an impossibility. The Keller-Segel model has shown good agreement with experimental data in prior work, see for example (22), so it is possible that further characterization of each strain variant combined with a more comprehensive exploration of variations of the Keller-Segel model may identify key strain characteristics and model parameters that account for the observed variation of band speeds.

Connecting single-cell properties with collective behavior is a necessary stepping stone for synthetic biology [66, 67]. Wild strains that demonstrate complex behaviors, such as the formation of a swarm band, are influenced by a combination of single-cell properties. Some properties are more dominant in setting collective behaviors [12, 21]. In the Ecc strains characterized here, the combination of run speed and tumbling frequency was sufficient to explain the overall band speed (Fig 4). Although, as shown in Fig 5, some strains have evolved different combinations of tumbling frequency and run speed to achieve the same band speed. In engineering collective behaviors of cellular networks, it may be possible to take advantage of different combinations of properties to achieve the same behavior, or it may be more efficient to build off of an existing combination of properties found in the wild. Each property likely has consequences on other cell behaviors, enabling simultaneous optimization of multiple phenotypes. Although, in this context, we only examined a simplified system with 7 wild isolates of the *Enterobacter cloacae* complex and 4 most-likely related strain phenotypes, introducing multiple phenotypic states into bacterial populations may be a useful approach to engineer other collective properties [37, 66, 68, 69]. Our results highlight the importance of comparing the characteristics of similar strains to untangle the connections between single-cell and collective behaviors. Environmental isolates are a good natural source of such variability and should serve to deepen our understanding of complex cellular phenotypes.

## Experimental procedures

### Migration screening

Enterobacter strains used in this study are listed in Table B in S1 File. Ecc1 was a member of a collection of wild isolates in a previous study [70]. To search for additional isolates that can form similar migration patterns, freshwater samples were collected from lakes and ponds in Los Angeles County. For isolation, samples were grown on HardyCHROM^™^ ECC selection plates. Six of these colonies were selected and identified to form a swarm band. After re-streaking the colonies on a fresh LB plate, the strains of interest were grown in LB media overnight and stored as frozen glycerol stocks at -80°C. 16S rRNA sequencing revealed the strain to be most closely related to members of the *Enterobacter cloacae* complex (Table B in S1 File).

For assessing each strain’s migration pattern, strains were inoculated from frozen glycerol stocks and grown to saturation overnight in M9 minimal salts (BD) supplemented with 2mM MgSO_4_, 0.1mM CaCl_2_, and 0.25% glycerol as the carbon source, as in [71]. Incubation condition was 200 rpm orbital shaking at 37°C Celsius. The following day, stationary phase cultures were diluted to an OD_600_ of 0.2. 10μL of this culture (about 10^6^ cells) was inoculated on the migration medium at this density for each experiment.

### Band visualization

Strains migrated on 4-well Rectangular plates (Nunc; ThermoScientific) at 25°C. Each well’s inner dimensions measured 80mm by 30mm, containing 6mL of migration media in semisolid agar, whose thickness is about 2.5 mm after settling. The migration media consisted of 0.26% agar, and M9 minimal salts (BD), 2mM MgSO_4_, 0.1mM CaCl_2_, 22mM (0.4%) glucose, 3mM sodium succinate, and 20μg/mL (0.002%) each of the amino acids histidine (0.13mM), methionine (0.13mM), threonine (0.17mM), and leucine (0.15mM) as previously utilized in similar experiments [24]. Components were excluded as noted. The liquefied gel, after being poured, was allowed to set on the benchtop for 1 hour. Then, at the lateral side of the major axis of the well, 10μL of bacteria culture at 0.2 OD_600_ was inoculated, following previous procedures [5, 23, 26]. Plates were lidded and sealed on all sides with parafilm to slow evaporation. To track the progress of the migration, each plate was mounted right-side up inside an enclosed imaging apparatus which held 4 plates at a time. The sole source of illumination were two white LED light fixture placed bilaterally on the same level as the plates. To avoid image aberrations from the lid, the camera (HD Pro C920; Logitech) imaged plates from the bottom. Time-lapse images over several days were recorded (VideoVelocity3; CandyLabs).

Changes in the bacterial cell density were shown as whiter pixels due to the contrast against a black background (Fig 1A). The position of the band could be easily tracked manually on ImageJ without further image processing. Coordinates of the band were measured at the center of band. Speeds of the bands were obtained by applying linear regression on the positions of the bands at 30 minute intervals and extracting the slope. Slopes were calculated for positions between 1.5 cm to 6 cm from the inoculation point to reduce boundary effects at the edge of the well.

### Tracking single-cell trajectories

In order to associate strain swimming properties at exponential phase to the band velocity observations, videos of cells in liquid media in a microfluidic device were obtained and analyzed for their trajectory features. Trajectories were not monitored during band propagation due to difficulty in obtaining long, single-cell trajectories within the band. Cells were grown overnight at 25°C in migration media described above without agar with orbital shaking at 200 rpm and diluted the next day 100-fold in fresh migration media. Cells were gently pipetted and not subject to vortexing in order to reduce flagella shearing. Cells were harvested at early exponential phase (3–4 hrs) and diluted again with fresh migration media to obtain tens of cells were field of view. The number of cells on screen (about a 0.15 mm^2^ area) was kept at about 50 cells to discourage cells from overlapping each other. This culture was injected into a 20μm high microfluidic chamber in a C-Chip (DHC-S02-2; INCYTO) and subsequently sealed with wax. The thin, sealed chamber was used to prevent directional fluid drift and to restrict the trajectories in a quasi-2D plane [17]. Before imaging, cells were incubated in the channel for at least 5 minutes to adapt to the fresh media. To avoid catching interaction of the swimming cells with the walls of the device, cells in the device were imaged as far away as possible (10μm) from the upper and lower walls of the chamber. Videos at 10 fps with a 40X Objective were taken for 5 minutes with an inverted phase-contrast microscope (Ti Eclipse; Nikon). At least 3 videos were taken at different areas of the chamber. A rolling paraboloid was used to accentuate the position of each cell by lowering the intensity of the background. Cell trajectories were obtained using the ImageJ plugin TrackMate, which detects positions of cells using a Gaussian profile fitter and subsequently forms links between spots that are proximal in position [35].

### Trajectory feature detection

Feature detection of runs and tumbles were performed following a previous study [34]. All trajectories shorter than 5 s were discarded. The percent of trajectories that were used for analysis after filtering for each strain was 20% to 33%, and the length of trajectories on average were 20 to 80 seconds long (Table C in S1 File). The time interval *Δt* of recorded positions is 0.1 s.

A tumble was defined as previously described based on sudden changes in direction [34]. We calculated the angular change *Δφ* at time *t* as the angle between two vectors around *t* (*t*‒*Δt* to *t* and *t* to *t+Δt*). We defined the time of local maximum angular change as *t*_*max*_ and the times of two neighboring minima as *t*_1_ and *t*_2_. If any angular change *Δφ* during *t*_2_ − *t*_1_ satisfied

$$|\Delta \varphi |>\gamma \sqrt{D_{r}(t_{2}-t_{1}),}$$ (1)

in which γ = 5 and *D*_*r*_ = 0.1 *rad*^2^*s*^−1^, the cell was identified as in turning state. Within the time interval, a tumbling event at time *t* was defined as when

$$\varphi (t_{max})-\varphi (t)\leq \;0.5\Delta \varphi \;,$$ (2)

where

$$\Delta \varphi \;=\;max[\varphi (t_{max})-\varphi (t_{1}),\varphi (t_{max})-\varphi (t_{2})].$$ (3)

By this definition, a trajectory segment with a smoothly curving path would not have tumbles associated with it.

Other studies report tumbling as either run time [59] or tumbling frequency [7]. After extracting these features from the trajectories in each video, distributions of each feature type were compiled for each strain. The tumbling frequency was then defined as the inverse of the average time length between two tumbling events. The run speed was calculated as the average speed of trajectory segments longer than 0.5 s between two successive tumbling events. The run time was calculated for all trajectory segments longer than 0.5 s. In calculating the average and standard deviation of the run time, data was fit to an exponential function to account for run times for run times than 0.5 s.

Swimming cells for each frame were counted by the number of cells in the frame with an average trajectory speed of at least 0.32 μm/s. The proportion of swimming cells in a frame was then calculated as the swimming cell number divided by the total number of trajectories in that frame. The proportion of swimming cells as a strain property was the average proportion of swimming cells per frame of the video.

### Growth curves

Cultures were grown in M9 media overnight, and the following day the cells in stationary phase were diluted to an OD_600_ of 0.01 in fresh M9 migration media. 200μL of each diluted culture was distributed in triplicates for each nutrient condition into 96 well plate (Costar). Edge wells were not used but rather were filled with water to prevent evaporation from the center wells. OD_600_ was read every 10 minutes at 25°C with 7 minutes of orbital shaking (Tecan M200; Tecan Group Ltd.). Exponential growth rates for each strain were obtained by considering an OD_600_ between 0.25 and 0.7 (between x and y cells for each strain), the range where the semilogarithmic plot of the growth curve was most linear. That means at least 10 absorbance measurements were used to calculate the growth rate. The experiment was repeated with the same settings for a total of 3 independent replicates. Plate counts were performed to calibrate absorbance measurements using the drop plate method [72].

### Multiple linear regression

Multiple linear regression has been widely used as a general approach for a variety of research problems [73–77]. The model for multiple linear regression, given the band speed as response variable and bacterial properties as predictor variables, is

$$BS\;=\;\beta_{0}+\beta_{1}RS+\beta_{2}TF+\beta_{3}PS+\beta_{4}GR,$$ (4)

where BS, RS, TF, PS, GR are band speed, run speed, tumbling frequency, proportion of swimming cells, and growth rate respectively. *β*_1_, *β*_2_, *β*_3_, *β*_4_ are regression coefficients of predictor variables, and *β*_0_ is the intercept.

The band speeds of all *Enterobacter* strains with and without methionine were analyzed, and corresponding bacterial properties into the design matrix. The equation can be written as

$$\begin{array}{cccc} \left[\begin{array}{c} BS_{1+}\\ BS_{1-}\\ \vdots \\ BS_{7+}\\ BS_{7-} \end{array}\right] & = & \left[\begin{array}{ccccc} 1 & RS_{1+} & TF_{1+} & PS_{1+} & GR_{1+}\\ 1 & RS_{1-} & TF_{1-} & PS_{1-} & GR_{1-}\\ \vdots & & \vdots \;\vdots & \vdots \;\vdots & \\ 1 & RS_{7+} & TF_{7+} & PS_{7+} & GR_{7+}\\ 1 & RS_{7-} & TF_{7-} & PS_{7-} & GR_{7-} \end{array}\right] & \left[\begin{array}{c} \beta_{0}\\ \beta_{1}\\ \beta_{2}\\ \beta_{3}\\ \beta_{4} \end{array}\right] \end{array}$$ (5)

with 1 to 7 stand for Ecc1 to Ecc7, + stands for with methionine, and—stands for without methionine. The regression coefficients and p-values were inferred using fitlm in MATLAB (MathWorks, Natick, MA).

The rescaled sensitivity was defined as the regression coefficient multiplied by the corresponding average measurement of all strains to make different properties comparable. For example, the rescaled sensitivity of run speed is

$$\beta_{1}\cdot mean({RS}_{1+},{RS}_{1-},\;.\;.\;.,{RS}_{7+},{RS}_{7-})$$ (6)

### 16S tree construction

For Ecc1 to Ecc6, genomic DNA was extracted using a DNeasy UltraClean Microbial Kit (Qiagen) and sequenced via Illumina HiSeq 2500 (UPC Genome Core; USC). The short reads were assembled by SPADES with a coverage depth of at least 100, and from the resulting contigs the 16S sequence was recovered [78]. For Ecc7, the 16S rRNA gene was amplified using colony PCR with the 8F and 1492R primers [79] and was sent for purification and Sanger sequencing (Laragen; Culver City). The 16S tree was constructed using base pairs 93 through 1436 of the 16S rRNA reference sequence of *E*. *coli* K-12 MG1655 on the online platform phylogeny.fr [80] and re-visualized using MATLAB’s Bioinformatics Toolbox. These strains were assigned to the *Enterobacter cloacae* complex by their similarity to *Enterobacter* strains using the EZBioCloud online platform [81] (Table B in S1 File). Ecc1 and Ecc4 seem to have identical 16S rRNA sequences over the analyzed region, although comparison of other genomic regions revealed these strains are not genetically identical.

## Statistical methods

To detect a monotonic relationship between two strain properties, a Spearman’s rho rank coefficient was computed [36].

## Supporting information

S1 File Includes Figs A-F, Tables A-C, and related text. (PDF)

S1 Movie Propagation of the Swarm Band in Semisolid Agar. Related to Fig 1. Growth of an inoculation of a strain and then a propagation of a band. Grayscale video is played back at 30 fps, where the time between each frame is 10 minutes. The band speed is taken from a linear regression of band positions between 1.5 cm and 6 cm from the site of inoculation.
(AVI)
