# Supplementary material for: Connecting single-cell properties to collective behavior in multiple wild isolates of the Enterobacter cloacae complex
Source: PLoS One. 2019 Apr 4;14(4):e0214719. doi: 10.1371/journal.pone.0214719 (PMC6448878; doi:10.1371/journal.pone.0214719)
Supplement: S1 File — (PDF) [file pone.0214719.s001.pdf]

Supporting Information for

Connecting single-cell properties to collective behavior in multiple wild isolates of the Enterobacter cloacae complex

Sean Lim, Xiaokan Guo, and James Q. Boedicker

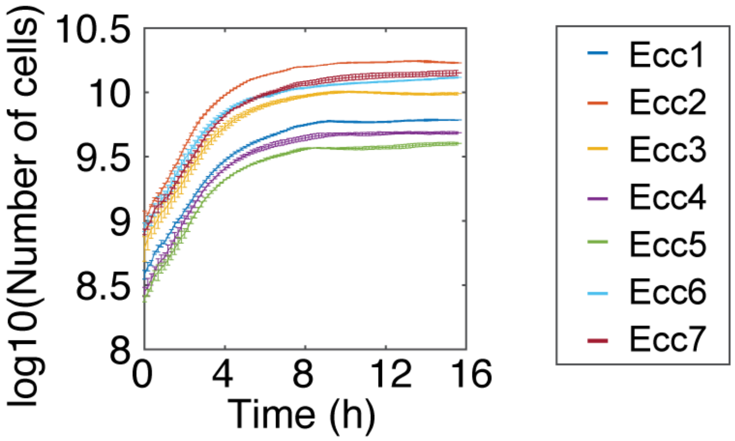

Fig. A: Growth curves of 7 *Enterobacter* strains. Related to Fig. 2E. Error bars represent standard error.

Cultures were grown in M9 media overnight, and the following day the cells in stationary phase were diluted to an OD_{600} of 0.01 in fresh M9 migration media. 200µL of each diluted culture was distributed in triplicates for each nutrient condition into 96 well plate (Costar). OD_{600} was read every 10 minutes at 25°C with 7 minutes of orbital shaking (Tecan M200; Tecan Group Ltd.).

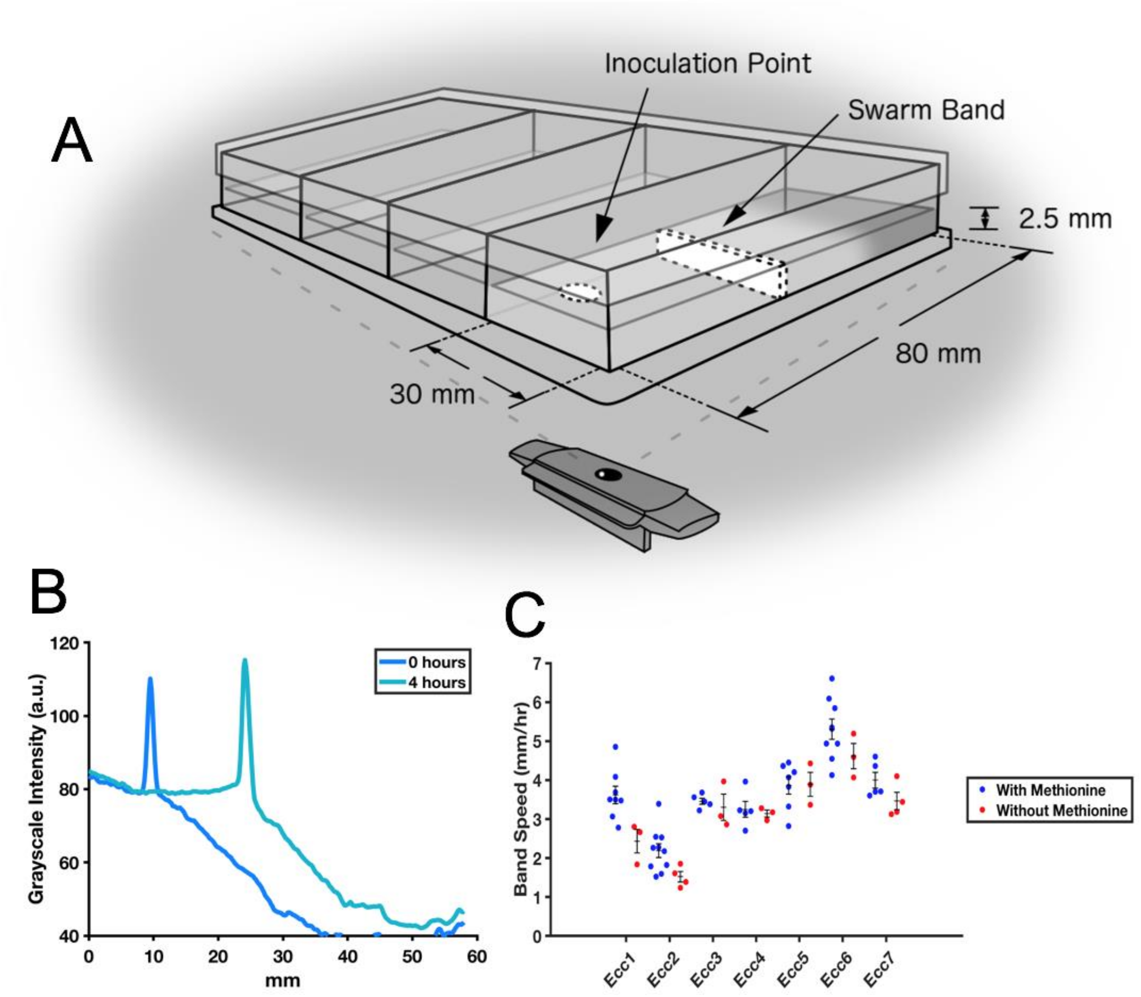

Fig. B: Swarm Band Detection and Speed Quantification. Related to Fig 1.

(A) Schematics of the imaging device used to take time-lapse images of the swarm band every 10 minutes. The webcam is fixed 22 cm below the imaging plane, where the plate sits. Light comes from the sides, illuminating the swarm band, which extends throughout the whole semisolid medium. Wells are separated such that multiple experiments could be run simultaneously. (B) A pixel intensity profile along the long axis of the rectangular well of two frames of a time-lapse of *Enterobacter* migration. The position of the band is taken at its peak and at the midline of the rectangular Petri dish. The band's peak can be clearly distinguished. (C) Band speeds measurements are shown as individual points, with error bars indicating the mean and SEM.

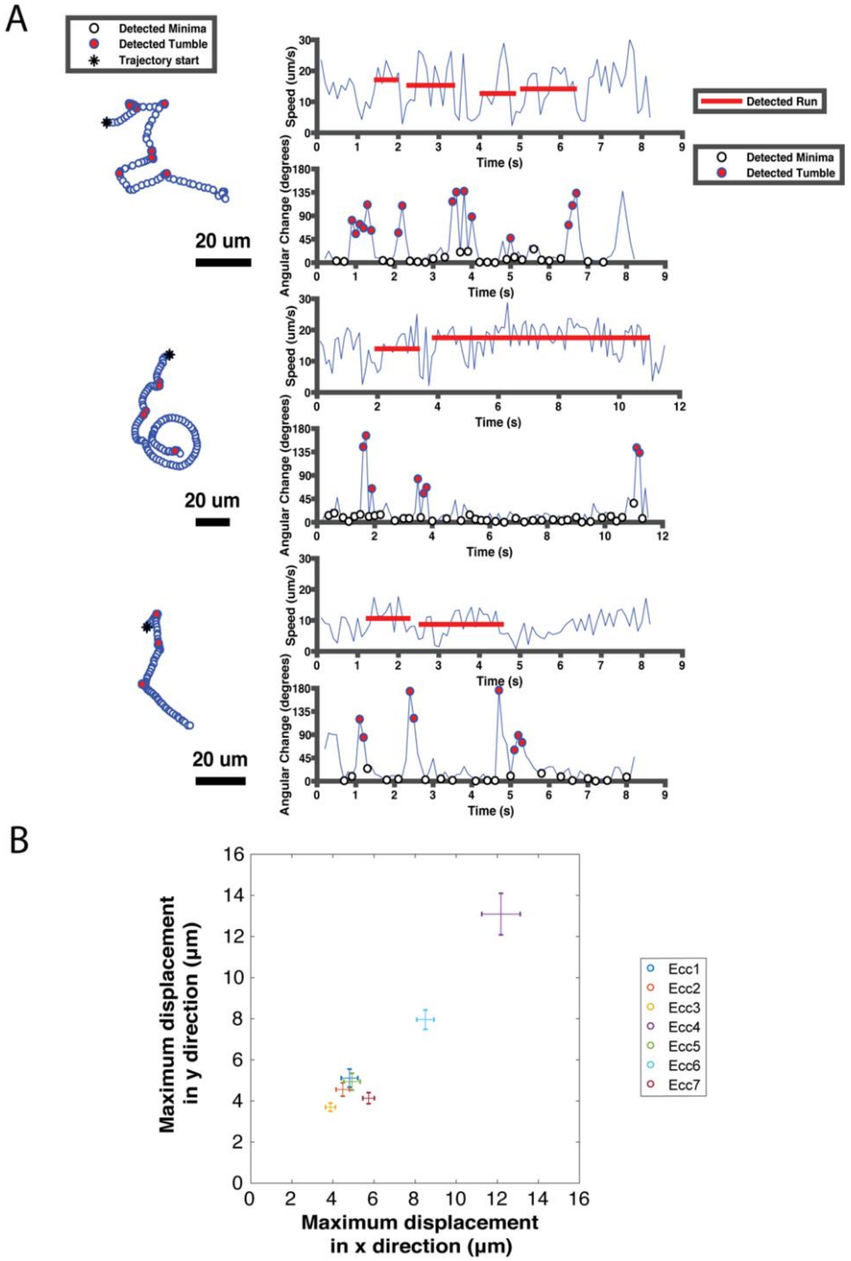

Fig. C: Trajectory Feature Detection. Related to Fig 2.

(A) Tumbles are detected by comparing different local maxima between two local minima of angular change along a trajectory. A run is detected between two tumbles. The amount of time between tumbles is the run time. The tumbling frequency is then calculated as the inverse of the average of the run time. (B) Average maximum displacement of non-motile/non-swimming cells over 3 minutes in x and y direction within the microfluidic chamber. Overall, the maximum displacement is longer than one cell length, indicating the non-motile cells are not stuck to the surface of the chamber. Analysis of the trajectories of these non-motile cells revealed an effective diffusion coefficient of 0.033 $\mu m^2/s$, consistent with a displacement of approximately 5 μm over 3 minutes.

A

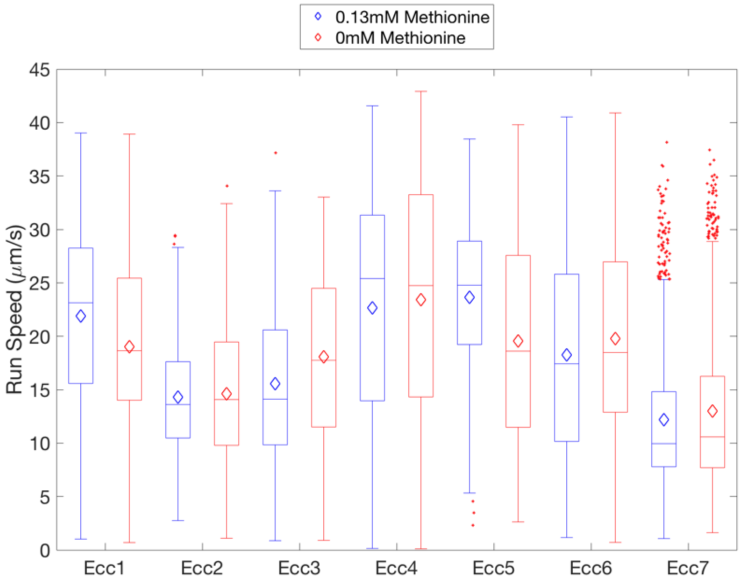

B

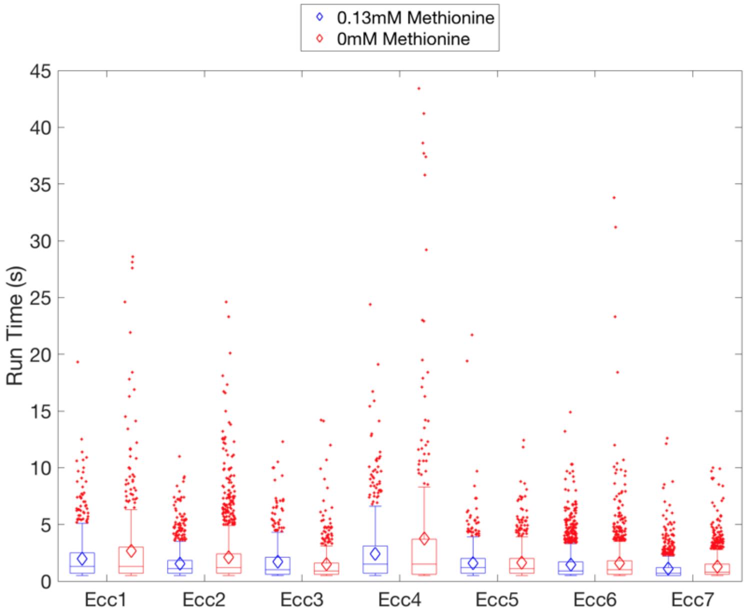

Fig. D: Run Speed and Run Time Distributions for Each Strain. Related to Figs 2 and 3.

(A) Run speed box plots of each strain for trajectories pooled from all 3 videos. Diamond = mean. Dots = outliers. Horizontal line = median. (B) Run time distributions of each strain. The inverse of the average run time is the tumbling frequency.

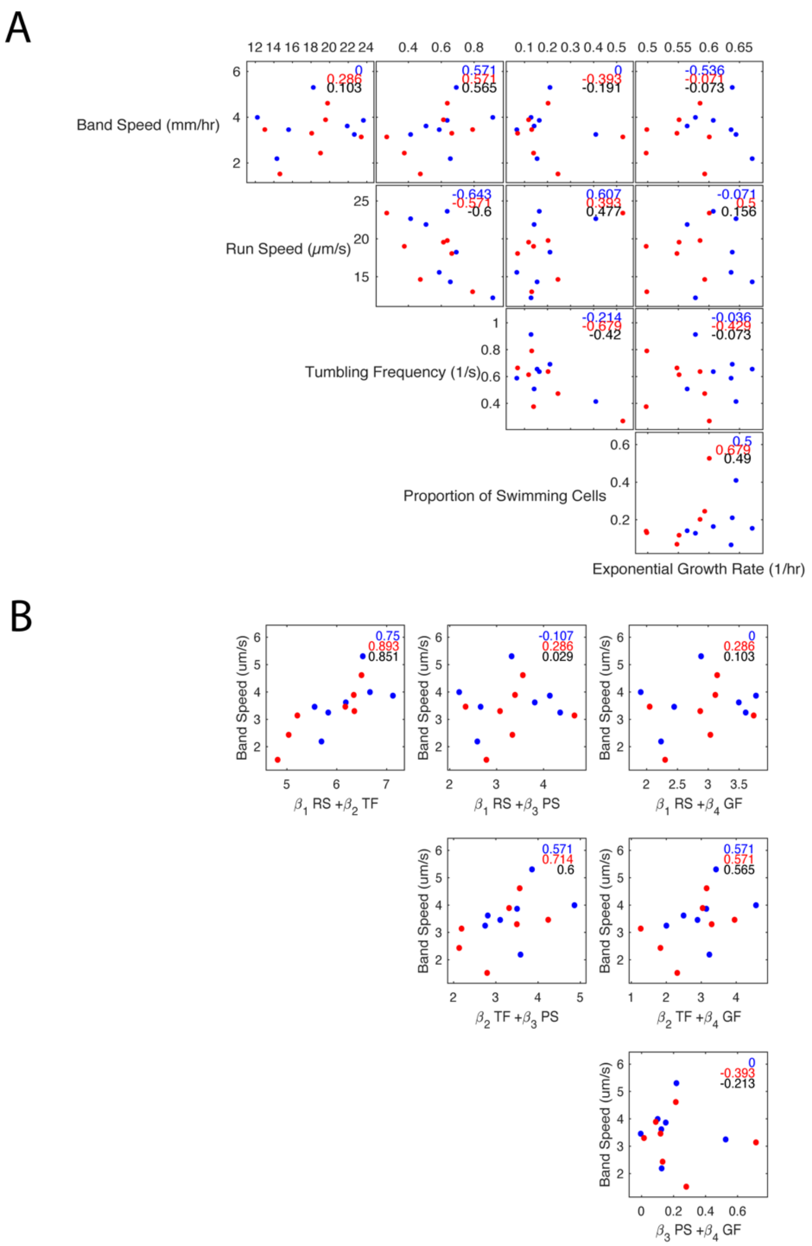

Fig. E: Pairwise Comparisons of Strain Properties with Band Speed. Related to Fig 4.
(A) All pairwise comparisons of averages of strain single-cell motility and growth properties. Data is grouped by the presence or absence of methionine. Numbers displayed are Spearman's rank coefficient (blue for with methionine dataset, red for without methionine dataset, and black for overall coefficient of combined datasets). The critical value for significance (p value < 0.05) is 0.786 for 7 samples and 0.538 for 14 samples. Only the combinations of band speed/tumbling frequency, and run speed/tumbling frequency (-/+ methionine combined) is above the threshold 0.538. This also suggests a negative correlation between the run speed and tumbling frequency. (B) Simultaneous correlations of two strain properties with coefficients found by Multiple Linear Regression with band speed. The combination of run speed and tumbling frequency has the strongest correlation with the band speed.

To theoretically predict the band speed from strain properties, we ran a simulation based on an extended Keller-Segel model that originally described in a similar study by Croze O. et al., in which bacterial cells grow and diffuse in porous media due to chemotaxis. The equations that describe the diffusions of bacterial cells and nutrients are

$$\frac{\partial b}{\partial t} = \mu_0 \mathrm{M(C)} \nabla^2 b - \chi_0 X(C) \nabla \cdot \left(b \frac{df_\chi(s)}{ds} \nabla \mathrm{s} \right) + \mathrm{b}(\mathrm{g(s)} - \frac{b}{k_b}), \tag{1}$$

$$\frac{\partial s}{\partial t} = \mathrm{D} \nabla^2 b - \frac{k_g}{Y} g(s) b, \tag{2}$$

where b and s are cell density and substrate concentration respectively, μ_0 is cell diffusivity, which is related to single-cell run speed v and tumbling frequency α_0 by $\mu_0 = \frac{v^2}{\alpha_0}$, χ_0 is chemotactic parameter, k_b is initial cell density, D is substrate diffusivity, k_g is growth rate, and Y is the bacterial yield defined by $b = Ys$. Also, $f_\chi(s) = \frac{s}{s+k_\chi}$, and $g(s) = \frac{s}{s+k_s}$, where k_χ is chemotactic threshold concentration, and k_s is growth threshold concentration. The dependence of bacterial diffusion on agar concentration C is described as

$$\mathrm{M(C)} = \frac{1}{1+f(C)}, \tag{3}$$

$$\mathrm{X(C)} = \frac{1}{\left(1+f(C)\right)^2} I_x(f(C)), \tag{4}$$

where $\mathrm{f(C)} = e^{\frac{C-C_1}{C_0}}$, in which C_0 and C_1 are characteristic concentrations, and

$$I_x\big(f(C)\big) = \frac{\int_0^\infty d\theta K(\theta) e^{-\alpha_0[1+f(C)]\theta}}{\int_0^\infty d\theta K(\theta) e^{-\alpha_0 \theta}}, \tag{5}$$

with

$$K(\theta) = N_0 e^{-\alpha_0 \theta} [1 - A_0 \left(\alpha_0 \theta + \frac{1}{2} {\alpha_0}^2 \theta^2 \right)], \tag{6}$$

where N_0 and A_0 are constants.

Using MATLAB, we run the numerical simulation in one-dimensional situation with no-flux boundary conditions

$$\mathrm{b(r,0)} = k_b e^{-\frac{k_g r^2}{\mu_0 \sigma^2}}, \tag{7}$$

$$\mathrm{s(r,0)} = \left(1 - s_0 e^{-\frac{k_g r^2}{\mu_0 \sigma^2}} \right), \tag{8}$$

where s_0 is the initial substrate concentration (of 4 amino acids), and σ is the width of initial inoculation point. Here we assume the added amino acids are the chemotactic substrate, although a chemotactic response towards glucose is also possible. The model here does not consider chemotaxis towards glucose. The simulations are run up to 8 hours with the band speed calculated as the slope of the band position over time from 3 to 8 hours, ignoring the initial non-linear part. The only parameters change for different strains are run speed v, tumbling frequency α_0, and growth rate k_g, as shown in Figs 2B, C, and E. All the other parameters used in the simulation are described in Table A.

Simulation were also run accounting for the small pore size of the gel. As examined in Licata at et al., 2016, the pore size, a, of agarose gels is estimated to be between 1 and 5 μm, similar to the size of a cell. The small pore size of the gel restricts cell movement, resulting in shorter runs with tumbling events leading to pore escape. Simulations were run replacing the cell diffusivity with $\mu_0 = a^2 \alpha_0$. This correction reduced

the cell diffusivity from approximately 1000 μm^2/s, in the limit of large pore size, to approximately 10 μm^2/s. The results of these simulations are shown in Fig. F panel B. Even after taking into account reduced cell movement due to the pore size of the gel, simulation results did not mirror overall trends in experimental data.

Parameter	Value	Source
μ_0	5.7 mm^2/h	Croze O. et al., 2011
χ_0	600 mm^2/h	Croze O. et al., 2011
k_b	3.5×10^8 cells/mL	Croze O. et al., 2011
D	3 mm^2/h	Croze O. et al., 2011
k_χ	0.5 mM	Croze O. et al., 2011
k_s	1 mM	Croze O. et al., 2011
Y	10^8 cells/mL/mM	Croze O. et al., 2011
C	0.26%	This study
C_0	0.035%	Croze O. et al., 2011
C_1	0.28%	Croze O. et al., 2011
A_0	0.5	Croze O. et al., 2011
s_0	0.58 mM	This study
σ	300	This study
a	5 μm	Licata et al., 2016

Table A: Parameter values used in the simulation.

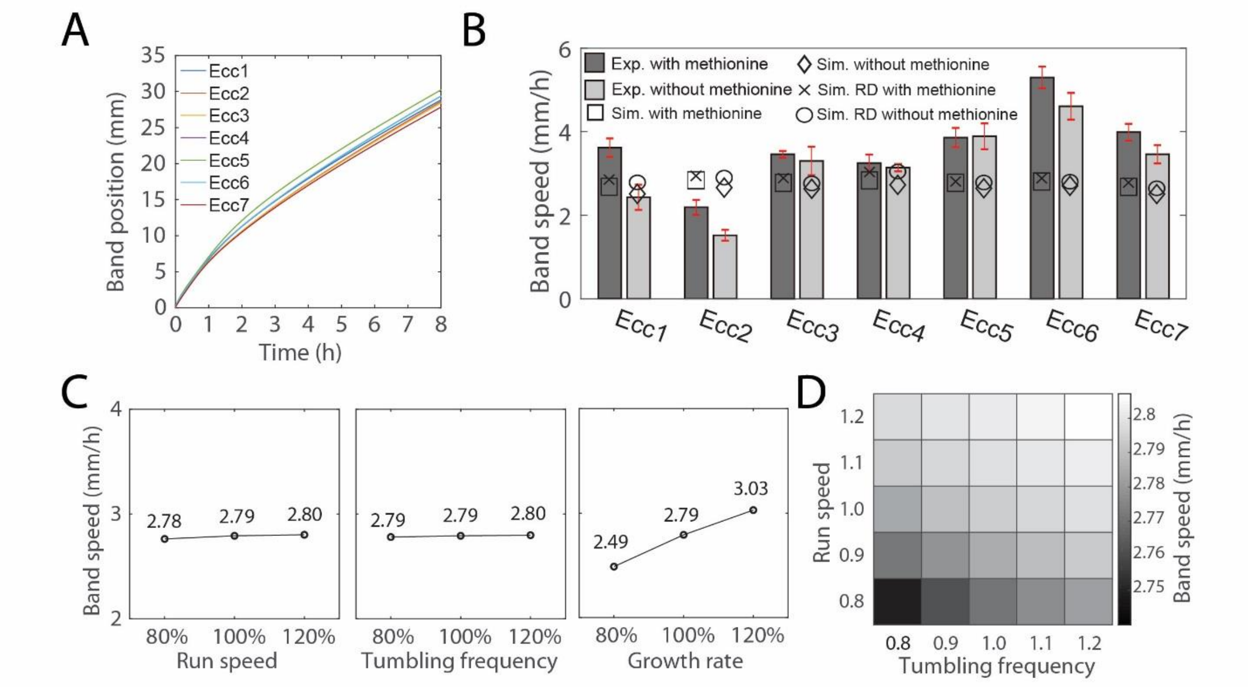

Fig. F: Theoretical prediction of the band speed from run speed, tumbling frequency, and growth rate using the Keller-Segel model. (A) The simulated migration of bacterial band over time for 7 *Enterobacter*

strains. (B) Simulated band speed in presence and absence of methionine for 7 *Enterobacter* strains, which shows similar trend as in the experiments. In an extension of the model, simulations which account for the restricted movement of the cells in a gel with small pores is shown as Sim. RD (simulation with restricted diffusion). (C) Run speed and tumbling frequency are positively and weakly correlated with the band speed. Growth rate is positively correlated with the band speed. 80%, 100%, and 120% of average value from 7 strains (with methionine) are used for each variable property. Fixed average values are used for the other two properties in each graph. (D) Heatmap shows run speed and tumbling frequency both positively contribute to the band speed. Simulations for C and D did not include the correction for the small pore size of the gel.

Running simulations, we obtained position vs time plot for all 7 *Enterobacter* strains as shown in Fig F panel A. Even with distinct strain properties from the experiments, there is little difference between band speeds of different strains. Fig F panel B shows simulated band speed from strain properties in presence and absence of methionine (Fig 3B). Surprisingly, all band speeds decrease when methionine is removed from the media, which highly matches the experiment results (Fig 3A).

In addition, we used 80%, 100%, and 120% of the original average value of each property to examine the effect of run speed, tumbling frequency, and growth rate on the band speed. The simulations suggest both run speed and tumbling frequency are positively correlated with the band speed but with very limited influence. Growth rate is strongly correlated with the band speed, which, however, is not supported by our experiments (Figs 2E and 4). Fig F panel D also shows that both run speed and tumbling frequency positively contribute to the band speed (Fig 4A), but to a much smaller extent with a difference of only 0.1 mm/h.

**Strain Number**	**Closely Related Taxon**	**Strain Name**	**16S Sequence Similarity to Taxon**	**Origin**	**Geocoordinates**
Ecc1	*Enterobacter ludwigii*	EcUSC1	1340/1344	Razo-Mejia et al. 2014	n/a
Ecc2	*Enterobacter homaechei subsp. steigerwaltii*	EcUSC2	1343/1344	Caltech Turtle Pond, Pasadena, CA	34.13695180867859,-118.12518197782356
Ecc3	*Enterobacter ludwigii*	EcUSC3	1344/1344	Exposition Park Rose Garden, Los Angeles, CA	34.01715461439311,-118.28612565994263
Ecc4	*Enterobacter ludwigii*	EcUSC4	1340/1344	Huntington Gardens, Pasadena, CA	34.12535874635576,-118.11155643191762
Ecc5	*Enterobacter tabaci*	EcUSC5	1342/1344	Huntington Gardens, Pasadena, CA	34.12535874635576,-118.11155643191762
Ecc6	*Enterobacter tabaci*	EcUSC6	1342/1344	Huntington Gardens, Pasadena, CA	34.12522283194115,-118.11121542831802
Ecc7	*Enterobacter ludwigii*	EcUSC7	1339/1344	Caltech Turtle Pond, Pasadena, CA	34.13698791452894,-118.12505054958183

Table B: Bacterial strains used in this study, related to Experimental Procedures.

Strain Number	Methionine Condition	Percent Included Trajectories	Total Included Trajectories	Average Time of Included Trajectory (seconds)	Median Time of Included Trajectory (seconds)	Average Tumbles Per Trajectory	Median Tumbles Per Trajectory	Percent Included Trajectories With Tumbles
Ecc1	+[a]	23%	917	50.6	12.8	17	5	36%
	-[b]	24%	1009	49.8	15.3	15	4	28%
Ecc2	+	31%	711	49.1	12.6	16	8	46%
	-	33%	843	42.3	13.4	16	5	53%
Ecc3	+	32%	743	72.9	29.5	29	5	22%
	-	25%	512	80.1	33.2	22	6	27%
Ecc4	+	24%	1630	25.6	8.6	10	2	47%
	-	25%	1055	23.5	8.9	13	2	39%
Ecc5	+	22%	623	53.2	11.1	10	6	53%
	-	20%	655	66.0	19.5	13	6	40%
Ecc6	+	21%	1319	42.9	10.4	19	8	54%
	-	20%	1235	45.2	11.3	13	6	50%
Ecc7	+	24%	707	56.5	15.5	47	27	37%
	-	25%	1068	49.6	14.2	30	23	39%

[a] 0.13mM methionine
[b] 0mM methionine

Table C: Trajectory statistics, related to Experimental Procedures.
